# Hospital and Institutional News

**Published:** 1917-11-10

**Authors:** 


### HOSPITAL AND INSTITUTIONAL NEWS.

### NATIONAL RELIEF FUND: WANTED A MEMBER OF PARLIAMENT.

We direct our readers' attention to the second leading article in our present issue, for the conduct on several occasions of some of those administering the funds of what is now called the National Relief Fund, and their allowing incompetent persons, whose one idea seems to be that the Poor Law is the proper channel and means for them to employ, to insult and injure the self-respect of the wives and dependents of our warriors, and to debase and insult the warriors themselves when rendered incapable of providing their own maintenance or that of those dependent on them, has done an infinity of mischief. We have often denounced strongly this fatal blot on the administration of the National Relief Fund, which, to our knowledge, has so exasperated some of the contributors to that Fund that they have often wished personally to take in hand the members who have been entrusted with its administration, who are in fact responsible for the reiterated attempts to debase and insult, through the Poor Law, those without whose services and devotion the nation could not continue to exist. Will some member of Parliament help the working classes throughout England by demanding to know who is the responsible head of the National Relief Fund at present, and insisting upon a list of the names of those administering this Fund who are the supporters and enforcers of the policy and the real cause of the evils and wrongs we have indicated?

### A PORTABLE WAR HOSPITAL.

The portable hospital which is being built for Columbia University is said to combine the best features of British and French designs. Most of the buildings are made of wood, but the operating theatre, the kitchen, the laundry, and the garage are built of thin sheets of steel, made in sections which can be easily put together. The foundations are made of concrete. The walls of the wooden buildings are double, with an air-space between. It is said that one of these buildings can be put up in two days by eight men, and that the whole hospital, which has been designed for 500 patients, can be erected within three weeks at the utmost, or in even less time, if the conditions are favourable. The total cost is estimated to be about £60,000. This includes water, gas, and lighting, and a steam-heating plant. This works out at a cost of about £120 per bed.

### THE PROCEEDS OF "FRANCE'S DAY."

The Lord Mayor of London went in State to the French Embassy on Tuesday of last week to present a cheque for £166,500 to M. Paul Cambon, on behalf of the British Empire Fund for French wounded. This magnificent sum was collected throughout England as a result of the special effort known as "France's Day." At the same time Lady Gertrude Cochrane presented a cheque for £10,000 as a grant from the Scottish Branch of the Red Cross Society. The Lord Mayor and the Mansion House are synonyms on the Continent for generosity irrespective of politics to any nation in affliction, and one cannot doubt that this signal proof of the goodwill of the British public towards our sister-in-arms will further strengthen the already close bonds of sympathy existing between us. M. Cambon, in an eloquent reply, referred to the Red Cross as a living proof of patriotism. "The spectacle given us by the Red Cross," he said, "is a comforting one, for if our soldiers fight and die for our country at the Front, our two nations behind the Front are fraternally united and work in harmony for the country." The Ambassador mentioned that the committee of the French Red Cross is now concerned with the establishment of hospitals for the treatment of soldiers who have contracted tuberculosis either in the trenches or on the sodden Flemish plains.

### NATIONAL SERVICE MEDICAL EXAMINATION.

At long last the promised guide to members of medical boards engaged in examining men for National Service is announced as being ready for issue. That some such publication was necessary in order to secure uniformity of procedure is evident. In the old recruiting days the medical officers were beset by a multitude of instructions, often contradictory, and little was done to secure a constant standard of classification being maintained. Under the present scheme all recruits will be seen at some period of the examination by each member of the board. As a rule the first examiner will take the height and weight, and note other physical characteristics; the second will note muscular development, deformities of joints, limbs, etc.; the third will test the organs of special sense; and the fourth will examine the chest and abdomen, and take the history of previous diseases. Each will initial the medical history sheet, indicating the grade for which he considers the man fitted. The final grading will be done by the chairman, who, in cases of doubt, will consult with the full board, and where the vote is equally divided give the casting vote himself. By this means something approaching to a uniform classification should be obtained.

### THE SOUTH AFRICAN AMBULANCE AT CANNES.

Not the least interesting of war hospitals across the Channel is that organised by the French Society of Cape Town and supported by contributions from South Africa. Situated on the sunny Riviera, in the Hôtel Beau Rivage, Cannes, the patients who are fortunate enough to be sent to it enjoy its delicious climate, which no doubt plays no small part in the curative process. Working in conjunction with the French Red Cross, its accessibility from the south-eastern section has brought to it many of the gallant defenders of Verdun, and the able work of its Surgeon-in-Chief, Lieut.-Col. Casalis, of the South African Medical Corps, has met with widespread recognition. It was recently described by General Champel, the officer entrusted by the French War Office with the task of inspecting military hospitals in France, as the finest he had visited. The cost of its maintenance is about £1,000 per month, part of which is defrayed by the French Government, but the Executive Committee now feel that the appeal should be broadened to include the British as well as the South African public. A work such as this, testifying to the sympathy existing between our Colonies and our brave comrades in arms, should not be hampered in any way by lack of funds.

### ORTHOPÆDIC WORK IN LEEDS.

To let the lay public into some of the mysteries of surgery and medicine is one of the surest ways of securing their sympathetic interest in the hospitals where they are being performed. A notable step in this direction was made by Lieut.-Colonel Littlewood, Chief Administrator of the Beckett's Park Hospital in Leeds, last week, at a luncheon-hour address to business men in that city. In simple, non-technical language he explained to them some of the newer methods of treatment now being adopted, and the tremendous advance that had been made in this branch of surgery since the war. "There is only one abiding source of happiness for humanity," he said, "and that is work." With this as his text, he went on to speak of the occupations that had been organised to occupy the men under his care during the tedious months of waiting, whilst severed nerves grew again and injured muscles regained activity. Sir Robert Jones it was who first suggested that the wounded would not only be far more resigned but it would materially assist the process of healing were they given useful work to do. At Beckett's Park this now includes basket-making, plumbing, tailoring, carpentry, weaving, and blacksmithing, and the men are, in consequence, far happier and more contented than in their days of idleness.

### THE GAY HOSPITALS OF BIRMINGHAM.

Since the outbreak of the war, through the Birmingham branches of the British Red Cross Society and the Navy League, hundreds of garden-parties, entertainments, and visits to theatres have been arranged in Birmingham, under the direction of the organising secretary of the Navy League, together with five or six special theatre matinées, at which from 1,000 to 3,000 wounded have attended. By this means about 100,000 wounded have been entertained, and the work has grown to such an extent that a Citizens' Central Organisation has been formed specially for this work by Alderman Neville Chamberlain, J.P., during his last few weeks as Lord Mayor of Birmingham. An organisation has been formed, known as the Birmingham Professions and General Trades Fund, of which Mr. J. H. Francis is chairman. So far in various ways they have entertained 87,317. One of the largest entertainments was the reproduction of the Alexandra Theatre pantomime—"Babes in the Wood"—at the Hollymoor War Hospital, when 100 performers took part. Almost weekly a performance is given from the Theatre Royal or Alexandra Theatre. At some of the hospitals entertainments are provided weekly from the Grand Theatre, Empire Palace, Birmingham Hippodrome, the Gaiety Theatre, and, we note with relief, the Repertory Theatre. The arrangements are carried out by a sub-committee, of which Mr. Philip Rodway is chairman. Some ten or twelve amateur concerts are given at the local hospitals each week, and during the summer months every hospital in Birmingham, and almost every hospital within twenty-five miles of Birmingham, has enjoyed a garden-party, when a good programme of prize sports has been arranged.

### A RAY OF HOPE FOR POOR-LAW REFORMERS.

Whether realised or not, the proposal for the amalgamation of the Petworth and Wisborough Green workhouses is more important to students of the Poor Law than it is probably felt to be by those immediately concerned. The suggestion for this amalgamation in Sussex is one of many similar suggestions, and the motive is the common desire to save expense to the ratepayers. This is a point which it is not worth while to discuss. The effect of the proposed amalgamation, here as elsewhere, is the point of larger interest. The best way to level up the Poor-Law infirmaries, to abolish lay control in the sick wards of workhouses, to provide adequately paid and skilled medical supervision, has been felt to be centralisation. Such far-sighted and experienced authorities as Dr. C. Thackray Parsons, if we may continue to give him his civilian title, have argued the case for centralisation before now; and beyond the difficulties which attend any change for the better anywhere, no serious fault in the reasoning, and therefore in the possibility of effecting this reform, has been found. For those who favour it, the economy campaign, under which the Unions are considering if they cannot pool their staffs, is worth watching. It may lead to nothing after the war. The bad old system may return. But it is permissible to hope that a desire to save the rates may, for once, set going a movement in the right direction, and prepare the way for an important institutional reform.

### SANATORIA AND PUBLIC SPIRIT.

The shortage of sanatorium beds grows worse rather than better, and it is with no feeling of satisfaction that the Liverpool Hospital for Consumption and the affiliated sanatorium at Kingswood, Delamere Forest, report "a very heavy list of patients waiting for sanatorium treatment." Mr. F. W. Young lately explained that the waiting list on the women's side was especially heavy, but apparently he thought it useless to consider any increase of beds at present. Is it really useless? The difficulties are obvious and immense, but sanatorium patients are better adapted for temporary buildings than any other class, and such buildings are of course, even at present, less difficult to obtain than any other. The financial difficulty is also felt at Liverpool, but, bluntly put, financial difficulties yield more easily to brains and energy than any other. In short, the position of any sanatorium with such a waiting list as that of Kingswood, even in war-time, is this: that it must show cause why it has so far felt unable to increase its accommodation. Anything short of a "defence" of this character will be felt to be unconvincing to competent and public-spirited observers.

### RECORD RAIN AND RECORD HEALTH.

During summer people loudly complain if it rains, but some statistics certainly seem to show that rain at that time of year is exceptionally good for the health of the community. Dr. Henry Malet, the medical officer of health for Wolverhampton, refers to this subject in his report for the quarter ending September 29. The summer weather was very unseasonable. The temperature was much below the average. Although generally warm there was little actual summer heat, and even this was modified by the almost incessant rain. Nevertheless, the death-rate, only 8.9 per thousand, was absolutely the minimum record. There was no cold to be injurious and no heat to cause summer diseases, while the cleansing effect of the enormous rainfall had been most valuable.

### THE TECHNIQUE OF THE ORDERLY.

The writings of Ward Muir on hospital life are so free from affectation or pious concealment, and so full of good sense, that his colleagues on the staffs of his own and other war hospitals would surely be sorry to miss any of his articles. We therefore draw attention to his recent article in the *Spectator* on "Being in Khaki." That part of it which deals with the technique of an orderly is, alas! a sentence only: "As a hospital orderly I had acquired the technique of the housemaid," he says. This, surely, is an insufficient statement of those acquirements which make a good orderly. We invite the writer, with his usual frankness and good humour, to return again to this subject, which still remains to most in the region of chaff. Now a much-chaffed official is always an important person or he would not be a common butt, and since most orderlies can give as good as they get in repartee, and enjoy the joke, the whole technique required of them is worth such full treatment as Ward Muir could probably give to it; for his custom is to write out his experience, and not to trifle unduly with the facts. The housemaid in khaki needs a Boswell in brief.

### HYPNOTISM AND INFLAMMATION.

In an interesting article in the current number of the *Lancet*, Dr. J. A. Hadfield, Temporary Surgeon R.N., gives an account of some remarkable experiments conducted by him upon a shell-shock patient in Haslar Hospital. By suggesting to the patient in the hypnotic state that he had been burnt and at the same time touching his arm with the finger, he was enabled to produce a large and painful blister which was surrounded by a considerable area of inflammation, and formed large blebs full of fluid, having the typical appearance of a blister produced by heat. In another experiment he hypnotised the patient and then suggested that he had been burnt, but that he would feel no pain. In this case no blister formed, thus seeming to indicate that it was the suggestion of pain which produced the blister. He then actually burnt the hypnotised patient, suggesting on one occasion that he had been burnt and on awaking would feel pain, and on the second that the burn would be painless. In the first case a large blister formed surrounded by a considerable area of hyperæmia, was painful, and took some time to heal. In the second there was a blister filled with fluid, but only a thin red line of demarcation between it and the normal skin, there was no pain, and it healed rapidly. The conclusions to which these experiments point are of some importance. Hilton, in his classic work "Rest and Pain," long ago pointed out the effect of pain in retarding the process of healing, and suggested that the nerve which supplied the part should be severed. If a patient could be so hypnotised as to abolish pain it seems possible that repair might be greatly facilitated. Other pathological conditions such as pleurisy might, as Dr. Hadfield suggests, be aided in the same way. The experiments were all carried out in the presence of competent medical witnesses.

### SALARY OF AN ASSISTANT OFFICER.

Bermondsey Board of Guardians have received a request for an increase of salary from the assistant medical officer at their infirmary. He was appointed to the position on January 1 this year, his salary and allowances amounting to £450 a year, rising by annual increments of £25 to £500 a year. He maintained that his request was not unreasonable when the board considered that a newly qualified medical man received a guinea a day, with all found, as assistant or *locum tenens*, and, estimating emoluments at the rate of £150 a year, this worked out at £533 5s. per annum. Temporary medical officers in the Army were receiving 24s. a day, plus £60 bonus at the end of each year's service, and a ration allowance whilst on service. This worked out at well over £500 a year. In comparison to these his position left much to be desired. Indoor men were assessed for income tax on their salaries alone, whereas he was assessed on his allowances as well, and he had the further disadvantage of having to reside in Bermondsey, where rents and rates were high. He added that his request was made solely on account of the increased cost of living, apart from any extra work entailed by the absence of some members of the permanent medical staff. As a result of his appeal, the board decided, with the sanction of the Local Government Board, to increase the salary from £450 to £500 a year from November 1.

### "RECALLED TO LIFE."

In the second number of the journal under the above name, which is devoted to the care, re-education, and return to civil life of disabled sailors and soldiers, the editor, Lord Charnwood, attempts, in a general survey, a medical classification of the disabled men so far discharged from the Navy and

Army during the war. As regards a rough division between injuries and diseases, the numbers are respectively 453 and 547 per thousand. Working under the same ratio it is estimated, to quote some headings, that there are 32 cases of injury to eyes, including an occasional case of total blindness, 30 of amputation of leg, 19 of arm (double amputation of limbs are not noted), 124 men with diseases of the chest, 50 rheumatic cases, 110 with diseases of the heart, 11 with epilepsy, 9 insane, 26 deaf, and 10 with frostbite. The classification, which is necessarily rough, will afford a general view of the problem, and give some idea of the variety of cases for which a Local War Pensions Committee may be required to find treatment. Lord Charnwood takes a cheerful view in expressing the opinion that the vast majority of discharged men do not leave the Navy or Army definitely marked as subjects for any special kind of care. The greater number are on the way to become fit for most purposes of life, with little more need of medical attendance than is common with civilians a little older than themselves, though often the completion of this recovery will involve persistence in some treatment which was recommended on their discharge.

### THE DONCASTER INFIRMARY.

The scheme for the rebuilding of the Doncaster Infirmary has been deferred until after the war. A deputation of the General Committee recently met representatives of the principal coal interests in the district served by the infirmary and discussed the position in regard to Miss Beckett Denison's bequest. While ready to accept substantial responsibilities in connection with the erection of a commodious and modern hospital, the coal-owners expressed the view that the present was an inopportune time for carrying the scheme through, and suggested that it be postponed until the end of the war. To this the General Committee agreed, and their decision was confirmed at the annual meeting of the governors on October 29. Grateful reference was also made at the annual meeting to the offer by Major and Mrs. Peake, of Bawtry Hall, of £10,000 towards the cost of the new hospital, as a memorial to their son Raymond, who was killed in the war; and the proposal of the Doncaster Co-operative Society to signalise the society's jubilee next year by a gift of £3,000 for an operating theatre was also accepted with hearty thanks.

### THE WELSH NATIONAL MEMORIAL ASSOCIATION.

When the Council of the Welsh National Memorial Association met at Llandrindod Wells on October 26, one of the chief matters which came before it was consideration of the position brought about by the resignation, owing to ill-health, of Dr. Marcus Paterson, the medical director. Great regret was expressed at Dr. Paterson's decision, and a resolution placing on record the Association's appreciation of his services was passed with acclamation. Major David Davies, M.P., who presided, said that Dr. Paterson had shared all the difficulties through which the Association had passed. In acknowledging the vote Dr. Paterson said he hoped the foundations he had helped to lay would prove both sound and lasting. In spite of objections by the Medical Committee, a recommendation of the Finance Committee that the vacant post be not filled during the war was adopted. The salary saved in this way will pass into the general fund.

### CHAPLAINS' SALARIES

A point of more than local interest was raised when strong protests were made at the Nottingham Board of Guardians on October 30 on a resolution to consider in committee a proposal that the salary of the chaplain, Rev. Canon Field, should be increased from £150 to £165 a year, and that of the Roman Catholic instructor, Rev. J. McIlroy, from £50 to £60. It was contended that this was a new departure, and that to apply such a policy of secrecy in regard to discussions on the salaries of all officials was going too far. In reply to these objections it was urged that there was nothing unfair about the procedure, and there were advantages in talking the matters over privately. The proposal to take the discussion behind closed doors was passed by fourteen votes to eight, and it was subsequently announced that the increases had been sanctioned by nineteen votes against six. Though there may be good reasons for the course adopted in the instances under review, in general the more satisfactory policy is one of open and free discussion of matters which are apt to be viewed in a rather critical light by the outside public.

### THE ROYAL ALBERT INSTITUTION.

Lord Richard Cavendish, presiding on October 26 at the annual meeting of the Royal Albert Institution at Lancaster, spoke of the success which had attended their abandonment of the "rather expensive and troublesome" practice of electing patients. Since the war the selection of patients had been left to the Central Committee, whose decisions had been heartily approved. The meeting was quite satisfied with this arrangement, and left the matter to the Central Committee once again. The speaker mentioned that the institution had lost by death during the past year several prominent supporters, among whom he named Lord Muncaster, a vice-president for over half a century, and Dr. Purey Cust, who had been chairman of the York Committee for nearly forty years.

### HOSPITALS PROVIDED AT SHORT NOTICE.

It is possible to give an idea of the difficulties some municipal authorities encountered in providing war hospital accommodation by giving quotations from a report of Dr. E. W. Hope, the medical officer of health for Liverpool. In August 1914 the Fazakerley Hospital was placed at the disposal of the military authorities. The administration departments of the institution were so constructed that in a very short time and at small expense the accommodation for the patients could be almost doubled. In view of the obvious fact that further hospital accommodation would be needed, the military authorities were advised at the time to avail themselves of this facility either by permitting the Corporation to erect temporary pavilions or alternatively erecting such pavilions themselves. Neither of these courses was acceptable to the authorities, and unhappily neither was ever acted upon. At that time also the Fazakerley Sanatorium and the hospital at Sparrow Hall—both institutions greatly needed—were approaching completion. The structural character of the building at Sparrow Hall made it comparatively easy for the work to be continued, and the various Government departments concerned—namely, the Local Government Board, the War Office, the Treasury, and the Ministry of Munitions—agreed to facilities being given for its completion, and, to assist still further the military authorities, the Sparrow Hall Hospital was handed over to them. The interests of the civil population were set aside for the time being; but the military authorities undertook to vacate the hospital at one week's notice, stating that they could easily do this. The sanatorium, which was even more urgently needed, did not fare so happily. In the first place, the building was not so far advanced. Difficulties in regard to materials as well as to labour were placed in the way, and, although the building was within a few months of completion, unhappily all work ceased upon it, notwithstanding that there was a list of some four hundred patients awaiting admission, and among these were not only soldiers who had contracted tuberculosis whilst on service, but their wives and dependants among the civilian population.

### HOW THE CONTROVERSY ENDED.

It was pointed out that if these two buildings were completed, one of them could be handed over to meet the needs of the military authorities, and the other would then be available for the urgent and pressing needs of the civilian population, whose health interests were seriously prejudiced by the want of hospital accommodation. However, matters drifted on. Quite apart from the needs of the civil population, it became increasingly apparent that more hospital accommodation might be required for military purposes, and the offer of the Corporation either itself to provide additional hospital accommodation for military cases or to assist the military authorities in doing so upon sites already available at Fazakerley was rejected. The Engineer officer with whom a final decision appeared to rest stated that "they did not discuss matters with Corporations, but if the offer of a Corporation were not such as to commend itself, it was immediately turned down." This, observes Dr. Hope, is an unfortunate attitude. After much correspondence, many interviews, and protracted delays, the military authorities consented to remove the difficulties in the way of the completion of the sanatorium provided that it was to be exclusively used for military purposes. This course, in the absence of any better arrangement, was accepted, it being distinctly understood that tuberculous soldiers invalided from the Army on account of tuberculosis should receive the same consideration as soldiers invalided for other medical reasons, such as dysentery or injury. These proposals, however, failed to materialise, and ultimately, as the result of an interview between representatives of the Ministry of Munitions and representatives of the Hospitals Committee, leave was given to the latter to proceed with the sanatorium with a view to its use for its original purpose.

### DURBAN AS A MILITARY STATION.

Durban, the Natal seaport, is becoming a military hospital base for Mesopotamia as well as for German East Africa, and provision is being made on an extensive scale. The building of a large brick hospital on the Beach frontage, between Addington Hospital and the Marine Parade—an ideal site—has been begun, and a great effort is being made so that it shall be completed, fitted and furnished by the end of the present year. The need for the speedy completion of the work is urgent, as 4,000 men are expected to be then sent back from Mesopotamia to Durban as patients for treatment. Further blocks will be added to the hospital later. A convalescent hospital is to be established at Pietermaritzburg, the Natal capital, seventy miles inland and 2,200 feet above sea level. Additional hospital accommodation is to be provided also at Capetown. Being a convenient half-way house between England and the East, Durban has become an important military station during the war for soldiers in transit, and as many as 35,000 troops have been there at one time. They generally stay a week or two, and sometimes longer. The troops live under canvas at the Imperial Military Rest Camp, situated on the Marine Parade. Australian troops to and from England also call at intervals at Durban. The total expenditure on the additional hospital accommodation, convalescent home, and hutments at the Imperial Military Rest Camp is estimated to run to nearly £400,000. The scheme is an arrangement arrived at between the Imperial, Union, and Indian Governments.

### MANUAL TRAINING IN SHELL-SHOCK.

Miracles in the workshops is the only phrase which will adequately describe the almost unexpectedly successful results which have accrued from the establishment of manual training centres by the Military Hospitals Commission in various parts of Canada, especially as regards the cases of ex-soldiers suffering from shell-shock. In the woodwork department of the vocational training branch at Victoria, British Columbia, for instance, are men who are suffering from nerve-centre injuries which have affected their powers of concentration. The work upon which they have been engaged has been the means of restoring their normal mental keenness through persevering efforts to focus their thoughts on the matter in hand. In this manner they gradually improve their condition and ultimately entirely overcome their difficulty. This class of vocational training is regarded by the Hospitals Commission authorities as of great therapeutic value. By the way, the Commission is to erect a 600-bed convalescent hospital at Ottawa, a project with which McGill University will also be associated.

### TOMMY'S NOTE-PAPER AND OTHER SCHEMES.

We learn that the Birmingham Branch of the British Red Cross Society has started one or two new schemes for the wounded. The first provides men in local hospitals with stationery, printed with the name and address of the hospitals, together with envelopes and postcards. About half a million has been used by the wounded. This humble provision is a great help to the wounded, for when a man writes to his relatives saying that he has arrived in Birmingham, as a rule he forgets to mention the name of the hospital. This causes great inconvenience owing to the loss of letters. One of the branches' activities is to send wounded men discharged from the military hospitals away to Aberystwyth for change. Where a case is recommended by a local hospital or medical man these men go away for two, or three, or four weeks at a time, and greatly benefit. Lectures are to be given in the hospitals by High Commissioners or Agents-General of the Colonies. The first takes place on Tuesday, November 13, when the Hon. F. W. Young, Agent-General for South Australia, will deliver an address.

### REAL WAR-BABIES.

The "war-baby" myth has long been exposed, but now that the country has periodically to suffer bombardment from the skies when the Huns come by aeroplane and Zeppelin, it may safely be said that there are "war babies" born to the sound of guns. In Bethnal Green the Mayor has called the attention of the Public Health Committee to the question of the ill-effects of air-raids upon expectant mothers and their offspring. The committee has asked the medical officer to furnish at frequent intervals reports upon the condition of the mothers and infants concerned, with a view to obtaining data as to the injury alleged to be caused by fright resulting from air-raids. The committee has written to the Local Government Board inquiring whether it would approve of any reasonable expenditure incurred by the Council in taking measures for the protection of expectant mothers and their offspring from the consequences of fright due to gun-fire and explosions, including, if deemed necessary, the removal of such persons from the danger zone. Meanwhile the Council proposes to take action. It possesses old brewery premises in Hackney Road which were acquired for the maternity and infant welfare scheme, and arrangements are to be made to utilise the ground floor of the malt-house for the shelter of expectant mothers during air-raids.

### INDUSTRY AND MOTHERHOOD.

In the far-off days before the war the question of women's employment in relation to their capacity as potential or actual mothers was one which occupied the attention of many social investigators. The Fabian Society, to mention but one, published much literature upon the subject, and the conclusion arrived at was that a high infantile death-rate was the inevitable concomitant of the whole-time employment of their mothers. In the first part of a report issued by the "Women's Industrial Council," statistics have been collected which go to show that this generally accepted conclusion is a fallacy. "No definite relation between married women's employment and infantile mortality can be discerned. Burnley has an infantile mortality of 90 per cent. above that of Nelson, but the proportion of married women working is only slightly higher—namely 414 per thousand in the former as compared with 371. Burnley and Wigan, with high and nearly similar rates of infantile mortality, have respectively the highest (414) and the lowest but two (95) rates of married women occupied." In view of the enormous numbers of women now engaged in industry, many of whom will be extremely loth after the war to relinquish their employments, it is most essential that the conditions of their occupation shall be such as to be favourable to healthy motherhood. It is, therefore, desirable that the fullest information should be accessible to Care Committees, parents, and young expectant mothers as to what occupations are not injurious, so that a wise choice can be made. Faced as we are with a falling birth-rate, and with the death of so many of the flower of our manhood, it is essential that the life of every infant should be safeguarded by all the means in our power.

### THIS WEEK'S DRUG MARKET.

The upward tendency in the price of quinine which has been noticeable during the past few weeks is maintained, and it seems probable that the advance will continue in view of the statistical position of the drug; were it not for the fact that sales of quinine are under Government control prices would probably be much higher than they are at present. The downward movement in the prices of benzoic acid and sodium benzoate has received a check, and the value is now inclined upwards. Aspirin is in more plentiful supply, and there are sellers at a little below the price that has been ruling for the past few months. There is no further change in the price of phenacetin. Balsam tolu is again rather dearer and in fair demand. The upward tendency in the price of cream of tartar continues in face of the increasing scarcity of the drug. The price of antipyrin is not quite so firmly maintained. The good demand for honey continues, and there appears to be little probability that prices will decline to any material extent; in fact, in view of the demand for all sweetening agents, the likelihood is that the price of honey will be still higher. Supplies of potassium permanganate are still difficult to get, and extreme prices are asked for anything available.

### TO OUR READERS.

Contributions are specially invited from any of the readers of The Hospital. They should deal with topical subjects and news or incidents illustrating any department of hospital work, its progress or difficulties. They must be authenticated for the information of the Editor only. The minimum payment if published is 5s. There is no hard-and-fast rule as to space, but paragraphs of about twenty lines in length are preferred.

